# Immunohistochemical distribution of secretagogin in the mouse brain

**DOI:** 10.3389/fnana.2023.1224342

**Published:** 2023-08-30

**Authors:** Pablo G. Téllez de Meneses, Laura Pérez-Revuelta, Ángel Canal-Alonso, Carlos Hernández-Pérez, Teresa Cocho, Jorge Valero, Eduardo Weruaga, David Díaz, José R. Alonso

**Affiliations:** ^1^Institute for Neuroscience of Castile and Leon (INCyL), Universidad de Salamanca, Salamanca, Spain; ^2^Institute of Biomedical Research of Salamanca (IBSAL), Salamanca, Spain; ^3^Bioinformatics, Intelligent Systems and Educational Technology (BISITE) Research Group, Universidad de Salamanca, Salamanca, Spain

**Keywords:** calcium-binding protein, cerebral nuclei, olfactory bulb, basal ganglia, hypothalamus, amygdala

## Abstract

**Introduction:**

Calcium is essential for the correct functioning of the central nervous system, and calcium-binding proteins help to finely regulate its concentration. Whereas some calcium-binding proteins such as calmodulin are ubiquitous and are present in many cell types, others such as calbindin, calretinin, and parvalbumin are expressed in specific neuronal populations. Secretagogin belongs to this latter group and its distribution throughout the brain is only partially known. In the present work, the distribution of secretagogin-immunopositive cells was studied in the entire brain of healthy adult mice.

**Methods:**

Adult male C57BL/DBA mice aged between 5 and 7 months were used. Their whole brain was sectioned and used for immunohistochemistry. Specific neural populations were observed in different zones and nuclei identified according to Paxinos mouse brain atlas.

**Results:**

Labelled cells were found with a Golgi-like staining, allowing an excellent characterization of their dendritic and axonal arborizations. Many secretagogin-positive cells were observed along different encephalic regions, especially in the olfactory bulb, basal ganglia, and hypothalamus. Immunostained populations were very heterogenous in both size and distribution, as some nuclei presented labelling in their entire extension, but in others, only scattered cells were present.

**Discussion:**

Secretagogin can provide a more complete vision of calcium-buffering mechanisms in the brain, and can be a useful neuronal marker in different brain areas for specific populations.

## 1. Introduction

Calcium (Ca^2+^) is essential for life, as it is necessary for the formation of teeth and bones, muscular contractions, and nervous system functioning. Among the multiple functions of Ca^2+^ in the nervous system, probably the most important one is acting as a second messenger, allowing neurotransmitter release and thus synaptic transmission (Augustine et al., 1987). To accomplish its functions, Ca^2+^ levels must be very finely regulated. One of the mechanisms involved in Ca^2+^ concentration control is the so-called calcium-binding proteins, which bind Ca^2+^ with appropriate affinity and specificity to maintain its concentration at specific levels (Brini et al., 2013). These calcium-binding proteins can be classified in two different families regarding their function: Ca^2+^ sensors and Ca^2+^ buffers. The first ones translate the signal of an increase in Ca^2+^ concentration into different biochemical responses, mainly by a conformational change. The best-known example is calmodulin. The other ones, Ca^2+^ buffers, modulate Ca^2+^ signals, as they bind free ions to remove them from the cytoplasm when they reach harmful levels or to transmit the Ca^2+^ signal throughout the cell (Gifford et al., 2007). Some examples of Ca^2+^ buffers are parvalbumin (PV), calretinin (CR) and calbindin-D28k (CB). Even though this functional classification is still valid, some Ca^2+^ buffers might also have sensor functions (Schwaller, 2009).

The abundance and variety of calcium-binding proteins in the central nervous system (CNS) is surprising. Some of them are ubiquitous, like calmodulin, but others are exclusively expressed in specific neuronal populations. This latter group includes PV (Celio and Heizmann, 1981), CR (Rogers, 1987), CB (Jande et al., 1981), and neurocalcin (NC) (Terasawa et al., 1992), all members of a homologous family of Ca^2+^ binding receptors, with a domain called EF-hand, which is a helix-loop-helix motif where the ion binds (Nakayama and Kretsinger, 1994). These proteins have distinct functions in the CNS. For example, PV-positive interneurons in the hippocampus might be involved in social memory, as they present higher activity when mice meet for the first time than when they are familiar with each other (Deng et al., 2019). In the case of CR, GABAergic neurons expressing CR in the prefrontal cortex might modulate limbic activity and thus affect emotional and cognitive behavior (Saffari et al., 2019). Nowadays, the relationship between these proteins and different pathologies has also been extensively studied, including autism spectrum disorders (Adorjan et al., 2017) and Alzheimer’s disease (Zallo et al., 2018). Beyond the study of their function, these proteins have also been widely used for neuroanatomical studies (Celio, 1990; Résibois and Rogers, 1992; Weruaga et al., 2000; Alonso et al., 2001; Briñón et al., 2001), as their staining reveals not only the neuronal soma but also dendritic processes and long portions of the axon.

Secretagogin (SCGN) is a calcium-binding protein discovered in 2000 in the β-pancreatic cells from the islets of Langerhans (Wagner et al., 2000). It is a member of the sensor family of calcium-binding proteins, with six EF-hand domains and the ability to bind four Ca^2+^ ions simultaneously with a half maximal affinity of 25 μM approximately, which is a relatively low affinity compared with other calcium-binding proteins (Rogstam et al., 2007).

In 2001 SCGN was observed for the first time in human *post-mortem* tissues (Gartner et al., 2001), and in 2009, it was studied in the nervous system of mice and lemurs, both in adult and developing neurons (Mulder et al., 2009). Since its discovery, SCGN has been widely studied in the brain of different species. Some studies show SCGN distribution in the whole brain, for example in fishes (Basu et al., 2022), birds (Gáti et al., 2014) or rats (Maj et al., 2012), but most of them focus on a specific brain area. In addition to the studies mentioned above, SCGN expression has been analyzed in different species in the amygdala, (Mulder et al., 2010; Gyengesi et al., 2013; Hevesi et al., 2021), the olfactory bulb (Mulder et al., 2009; Kosaka and Kosaka, 2013; Ortiz-Leal et al., 2023), or the hippocampus (Attems et al., 2007; Tapia-González et al., 2020), among others.

However, the specific function of SCGN in the CNS is still unclear. Some authors suggest that it is involved in the release of stress hormone, as it is necessary for the secretion of adrenocorticotropic hormone (Romanov et al., 2015). At a cellular level, SCGN is expressed earlier than other calcium-binding proteins in migrating neurons, so it could play an important role in the formation of neuronal circuits (Mulder et al., 2010), and may be involved in processes such as neuronal remodeling, differentiation, and proliferation (Alpár et al., 2012).

According to pathological situations, the study of SCGN may be relevant in the Alzheimer’s disease field. SCGN might make neurons largely resistant to neurodegeneration, as in human hippocampus the colocalization between SCGN and Tau-positive neurons has rarely been observed (Attems et al., 2008). Moreover, in a transgenic mice model with a widespread Tau pathology, the expression of SCGN in the hippocampus is reduced (Attems et al., 2011) and it has been observed in rats that SCGN interacts with Tau protein (Maj et al., 2012). In addition to Alzheimer’s disease, a deficiency in SCGN might be a risk factor for autism spectrum disorders (Liu et al., 2023). It has recently been discovered that the interaction between SCGN and Doc2α (a protein related to autism spectrum disorders) plays an important role in neuronal morphology, glutamatergic transmission and different behaviors of mice, leading to abnormalities if such interaction is disrupted (Wang et al., 2023). Finally, it has also been observed that SCGN could help to compensate for sensory deprivation in the olfactory bulb of mice (Pérez-Revuelta et al., 2020). However, additional information is necessary to support or reject these hypotheses, and the anatomical distribution of this protein can provide useful clues.

A major peculiarity of the nervous tissue is its intrinsic heterogeneity. Whereas cells in other tissues are considered similar elements, neurons differ in their morphology, position, synaptic connections, and chemical content. The study of the expression of different proteins along the nervous system is a useful tool (Merkulyeva et al., 2016; Reuss et al., 2016), as knowing the specific distribution of proteins can lead to establishing their function. Regarding SCGN expression, it has revealed new cellular types in different regions and species. For instance, in the mouse main olfactory bulb, SCGN marks some juxtaglomerular cells that do not express other neurochemical classification markers (Mulder et al., 2009; Kosaka and Kosaka, 2013). Similarly, in the zebrafish retina, SCGN labels a novel subpopulation of amacrine interneurons (Dudczig et al., 2017). Finally, in the rat striatum, SCGN characterizes certain subpopulations of known striatal interneurons, but probably also other neuronal groups with unknown function and structure (Kosaka et al., 2017).

In conclusion, even though different research articles have reported SCGN distribution in some discrete regions of the CNS of healthy adult mice, a more exhaustive analysis is needed. Here, we present a detailed study of SCGN expression in the whole mouse brain using immunohistochemistry, which allows us to observe a characteristic distribution of immunopositive neurons.

## 2. Materials and methods

### 2.1. Tissue processing and immunohistochemistry

Four adult male C57BL/DBA mice aged between 5 and 7 months were used. The treatment of these animals was according to the European Union Directive (2010/63/UE) and the Spanish Current Law (RD118/2021) for the maintenance, use, and care of laboratory animals. The protocol for this study was approved by the Bioethics Committee of the University of Salamanca.

Under deep anesthesia, heparin was injected into the left ventricle to prevent blood clotting. Then, the right atrium was opened to allow blood flow and the mice were perfused with 0.9% (w/v) saline solution, followed by Somogyi’s fixative. This fixative is composed of 4% (w/v) paraformaldehyde and 15% (v/v) saturated picric acid in 0.2 M phosphate buffer (PB), pH 7.4. After 15 min, the brain was removed and left in Somogyi’s fixative for 2 h. Finally, the brain was cryoprotected in 30% (w/v) sucrose and sectioned either coronally or sagittally using a freezing sliding microtome (Leica Frigomobil, Jung SM 2000, Nussloch, Germany), obtaining 30 μm-thick slices. Sections for immunohistochemistry were washed 3 × 10 min in 0.1 M phosphate buffered-saline (PBS) pH 7.3 and incubated with primary antiserum. This antiserum was composed of 0.2% (v/v) Triton X-100 (Probus S.A., Barcelona, Spain), 5% (v/v) donkey serum (Cultek, Madrid, Spain), and 1:50 000 rabbit polyclonal antibody against SCGN, kindly provided by Wagner et al. (2000), in PBS. After 48 h, the slices were washed again 3 × 10 min in PBS and incubated for 1 h with the secondary antiserum. In this case, the antiserum was composed of 1:300 donkey biotinylated anti-IgG (Jackson ImmunoResearch, Cambridge, UK), in PBS. After this time, sections were washed 3 × 10 min in PBS and incubated with 1:200 avidin-biotin-peroxidase complex (Vector Laboratories, Inc., Burlingame, CA, USA) in PBS for 1 h in the dark. Finally, the samples were rinsed 3 × 10 min in PBS and 10 min more in 0.2 M Tris–HCl, pH 7.6, and revealed in a medium containing 0.003% (v/v) H_2_O_2_ and 0.02% (v/v) 3,3′-Diaminobenzidine (DAB; Sigma-Aldrich, San Luis, MO, USA) in 0.2 M Tris–HCl, pH 7.6. The reaction was controlled with a microscope and stopped by washing the sections with 0.2 M Tris–HCl, pH 7.6.

Specific controls were performed by removing either primary or secondary antibodies, and any non-specific staining was not detected.

Slices were mounted in gelatinized slides and dehydrated using alcohols of increasing alcohol content and xylol, before being covered with Entellan (Millipore Corporation, Burlington, MA, USA) and coverslips.

### 2.2. Image analysis

Images were obtained using a slide scanner microscope (Olympus VS200, Tokyo, Japan) coupled with a VS-264C digital camera. The Olympus OLyVIA software (Olympus) and the Fiji programme (Schindelin et al., 2012) were used to process the images. The stereotaxic atlas of the mouse brain (Paxinos and Franklin, 2001) and Allen Brain Atlas (Allen Institute for Brain Science, 2018) were used to identify different nuclei and structures.

Finally, we verified our semiquantitative description of the staining of the area occupied by SCGN-positive cell somata in each nucleus (showed in Table 1) with the Fiji threshold tool (Schindelin et al., 2012). In addition, some coronal diagrams based on Paxinos and Franklin (2001) with the relative number of positive somata were done for a better understanding of our results (Figure 8).

**TABLE 1:** Distribution of SCGN-positive cells in the mouse brain, referred to the area of each nucleus covered by cell somata.

Brain region	Cell amount	Figures	Additional remarks
**1. Olfactory bulb**			
1.1 Glomerular layer	++	1A,B	Juxtaglomerular neurons
1.2 External plexiform layer	+	1A	Intense fibre staining and few isolated somata
1.3 Mitral cell layer	++	1A	Line of strongly labelled somata
1.4 Internal plexiform layer	+	1A	Intense fibre staining and few isolated somata
1.5 Granule cell layer, external zone	+++	1A,C	Granule cells, irregularly distributed
1.6 Mitral cell layer of the accessory olfactory bulb	++	1D	
1.7 Granule cell layer of the accessory olfactory bulb	+++	1D	Granule cells
**2. Cerebral cortex**			
2.1 Tenia tecta	++	2A	Dense cell layer of the tenia tecta
2.2 Hippocampal formation			
*2.2.1 Granule cell layer of the dentated gyrus*	+++	2B	Small, rounded cells, only somata weakly labelled
*2.2.2 Fasciola cinereum*	++	2C	Fibres strongly labelled
*2.2.3 Indusium griseum*	+++	2D	Strong staining both in somata and neurites
**3. Basal ganglia and forebrain**			
3.1 Accumbens nucleus	+	3A	
3.2 Medial septal nucleus	+	–	Scattered isolated cells
3.3 Nucleus of the vertical limb of the diagonal band of Broca	+	–	Scattered isolated cells
3.4 Bed nucleus of the stria terminalis			
*3.4.1 Medial division*	+++	3B-D, 5C	Big cells with intense staining and small, rounded cells with faint staining. Homogenously distributed
*3.4.2 Lateral division*	++	3C	
3.5 Ventral pallidum	+	3D-F	Disperse cells. Large with intense staining and small with weaker staining. Some bipolar cells (3E) and multipolar cells (3F)
3.6 Interstitial nucleus of the posterior limb of the anterior commissure	+		
3.7 Nucleus of the horizontal limb of the diagonal band of Broca	+		
3.8 Substantia innominata	+		
3.9 Lateral preoptic area	+		
3.10 Magnocellular preoptic nucleus	+		
3.11 Medial preoptic area	++	5C	Many cells but faint staining
3.12 Medial preoptic nucleus	++	5C	Many cells but faint staining
3.13 Amygdala			
*3.13.1 Rostralmost part of the anterior part of the basomedial amygdaloid nucleus/dorsal part of the anterior amygdaloid area*	+	4A,B	Disperse cells, strongly labelled with big size and some of them with bipolar morphology (4B)
*3.13.2 Central amygdaloid nucleus*			
- 3.13.2.1 Medial division	+/++	4A,C,D	Disperse cells in the rostra part (4A), but small, densely packed with intense staining in the caudal part (4C,D)
- 3.13.2.2 Capsular part	++	4C,D	
- 3.13.2.3 Lateral division	+	4C,D	
*3.13.3 Medial amygdaloid nucleus*	++	4A,C,E	Small cells with weak staining and big ones strongly labelled
*3.13.4 Intraamygdaloid division of the bed nucleus of the stria terminalis*	+	4C	Scattered, weakly labelled
**4. Hypothalamus**			
4.1 Arcuate nucleus	+++	5A,D	Stronger staining in the somata than in the neurites
4.2 Paraventricular hypothalamic nucleus	+++	5B	Strong staining both in somata and neurites
4.3 Anterior hypothalamic area	++	5B,C	Faint staining
4.4 Ventromedial hypothalamic nucleus	+	5D	Very faint staining
4.5 Medial tuberal nucleus	+	5D	Faint staining
4.6 Dorsomedial hypothalamic nucleus	++	5D	Heterogeneous distribution, some areas with many cells and others with almost none.
4.7 Periventricular hypothalamic nucleus	+	5C	
4.8 Subincertal nucleus	+	5D	Weakly labelled
**5. Diencephalon**			
5.1 Thalamus			
*5.1.1 Dorsal lateral geniculate nucleus*	++	6A-D	Some bipolar cells
*5.1.2 Magnocellular part of the ventral lateral geniculate nucleus*	+	6A-C	Mainly close to the ventricle. Some bipolar cells
*5.1.3 Dorsal part of the lateral posterior thalamic nucleus*	+	6A	
5.2 Pretectal zone	++	6F	
5.3 Zona incerta	+	4C	Weakly labelled
**6. Mesencephalon**			
6.1 Superior colliculus			
*6.1.1 Superficial gray layer of the superior colliculus*	+	6E	
*6.1.2 Zonal layer of the superior colliculus*	++	6E	
*6.1.3 Optic nerve layer of the superior colliculus*	+	6E	
6.2 Substantia nigra	+	-	
**7. Brainstem and cerebellum**			
7.1 Cuneiform nucleus	++	7A	Dense group of cells with many strongly labelled neurites
7.2 Microcellular tegmental nucleus	++	7A	Mainly fibres and some cells
*7.3 Dorsolateral periaqueductal gray	++	7B	Only fibres
*7.4 Dorsomedial periaqueductal gray	+	7B	Only fibres
7.5 Nucleus of the solitary tract	+	7C	Big, faintly labelled cells, in the anterior part of the nucleus
7.6 Dorsal motor nucleus of the vagus	+	7D	Big, faintly labelled cells

Cell amount being low, + (approximately between 0.1 and 2.5% of the area covered); medium ++ (approximately between 2.5 and 10% of the area covered); and high, +++ (approximately between 10 and 20% of the area covered). *Only fibres were found.

## 3. Results

Secretagogin-immunoreactive cells were observed in many different nuclei and areas, with no differences among animals. The main characteristics of these groups of cells throughout the brain are described. A figure with coronal schemata of the more representative nuclei and areas with positive somata can be found at the end of the results (see Figure 8). Also, at the end of this part, a summary table collects all the nuclei presenting SCGN-positive cells, a semiquantitative analysis of the area covered by cell somata and the figures where they can be found (Table 1).

### 3.1. Olfactory bulb

**FIGURE 1:**
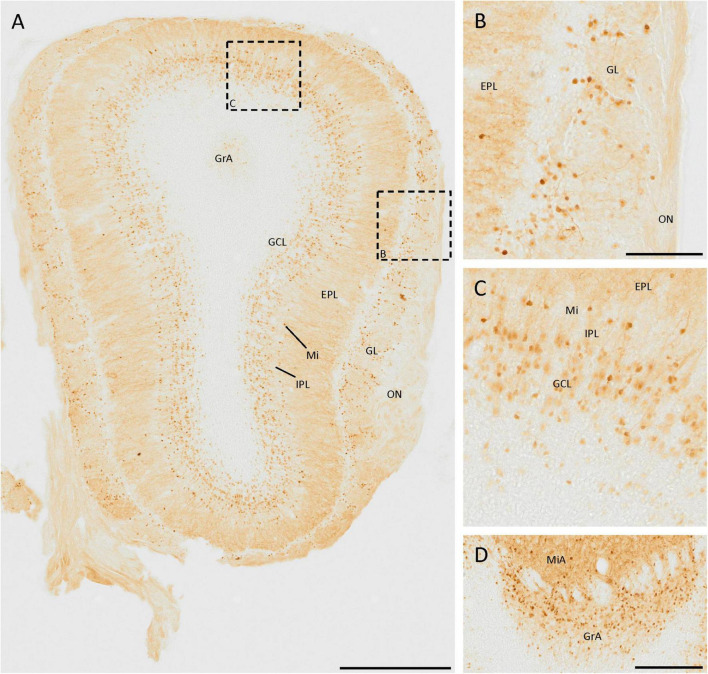
SCGN expression in the olfactory bulb. **(A)** General view of the olfactory bulb showing the high number of SCGN-positive cells. Note the number of stained fibers in the external plexiform layer (EPL) **(B)** Distribution of SCGN-positive cells in the glomerular layer (GL), surrounding the glomeruli. **(C)** Higher magnification of the mitral cell layer (Mi) and granule cell layer (GCL), where immunostained cells can be observed in both layers. **(D)** Positive cells in the mitral and granular cell layer of the accessory olfactory bulb (MiA and GrA, respectively), where the immunoreactivity is similar to that found in the Mi and GCL. IPL, internal plexiform layer; ON, olfactory nerve layer. Scale bar: **(A)** 500 μm; **(B,C)** 100 μm; **(D)** 200 μm.

The SCGN staining in the olfactory bulb has been studied in detail (Kosaka and Kosaka, 2013), and our results agree with those previously reported (Figure 1A). There are many small neurons in the glomerular layer, around the glomeruli, as they are juxtaglomerular neurons (Figures 1A, B). Deeper in the olfactory bulb, some isolated cells and intense fiber staining were observed in the external plexiform layer (Figure 1A). In the mitral cell layer, a line of strongly labeled cell somata was observed. The staining in the internal plexiform layer was similar to that found in the external plexiform layer. Finally, in the granule cell layer, many positive cells were detected, corresponding to granule cells. They were irregularly distributed, as the density of positive cells was higher in the external zone than in the innermost portion (Figures 1A, C).

In the mitral and granule cell layers of the accessory olfactory bulb, the same pattern of expression as in the mitral and granule cell layers of the main olfactory bulb was observed (Figure 1D).

### 3.2. Cerebral cortex

Calcium-binding proteins are usually abundant in the cerebral cortex, but this is not the case for SCGN, as few positive cells were spotted here, with very specific locations. Some isolated cells were found in the isocortex, but always in the proximity of the corpus callosum or the external capsule.

**FIGURE 2:**
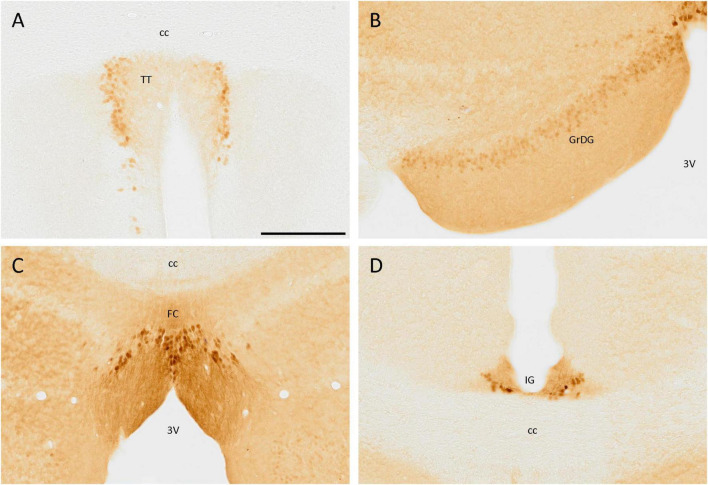
SCGN immunostaining in the cortex. **(A)** Cells positive for SCGN in the tenia tecta (TT) present a faint staining but a particular distribution, close to each other and with the fibers oriented toward the center. **(B)** The labeling in the granule cell layer of the dentate gyrus (GrDG) is weak, but defines the layer well, especially in the rostral slices. **(C)** Labeled cells in the fasciola cinereum (FC) had a characteristic distribution, similar to that found in the TT, but with stronger staining. **(D)** Cells in the indusium griseum (IG) were few and tended to be in the ventral part of the area, but the labeling was consistent along the cortex. 3V, third ventricle; cc, corpus callosum. Scale bar: 200 μm.

In the tenia tecta, a group of cells expressing SCGN was distributed in a vertical band, with its neurites orientated toward the central area, being part of the dense cell layer of the tenia tecta (Figure 2A). This distribution was present from Bregma 1.94 and caudally to this point, with only isolated cells in more rostral regions. In the most caudal part of this cortex, no more positive somata were observed, and only positive fibers were detected.

In the hippocampal formation, positive cells were distributed in the granule cell layer of the dentate gyrus, but the staining was faint. These cells were small and rounded and only somata were labeled (Figure 2B). Positive cells were also observed in the fasciola cinereum, forming a layer where the hippocampus begins and with their fibers strongly labeled and directed toward the third ventricle (Figure 2C). Finally, in the indusium griseum, despite its small size, a few cells with intense staining in both soma and neurites were observed (Figure 2D).

### 3.3. Basal ganglia and forebrain

**FIGURE 3:**
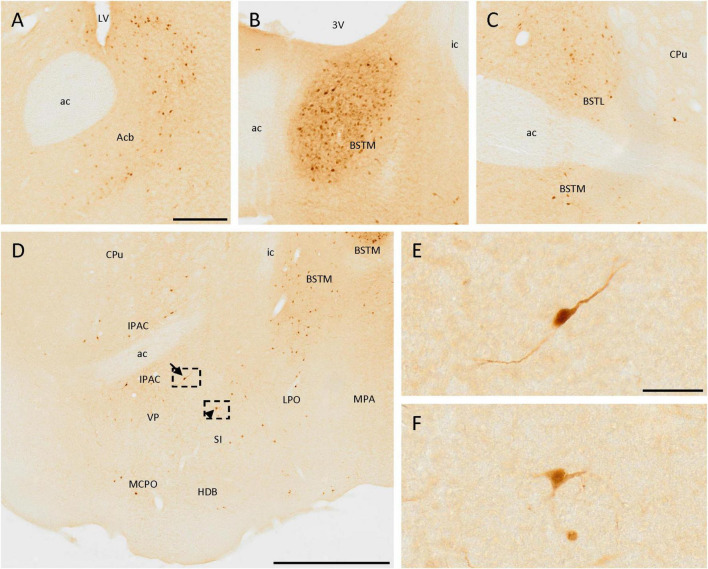
SCGN expression in the basal ganglia. **(A)** Positive cells in the accumbens nucleus (Acb) surrounding the medial part of the anterior commissure (ac). **(B)** Group of stained cells in the medial division of the bed nucleus of the stria terminalis (BSTM). **(C)** In the lateral division of the bed nucleus of the stria terminalis (BSTL) cells are similar to that found in the BSTM, but the density of positive cells is lower. **(D)** Positive cells in different nuclei from the basal ganglia showing different neuronal morphologies, such as bipolar (arrow) and multipolar (arrowhead). **(E)** Detail of the bipolar cell pointed with the arrow in panel **(D)**. **(F)** Detail of the multipolar cell pointed with the arrowhead in panel **(D)**. 3V, third ventricle; ac, anterior commissure; CPu, caudate putamen (striatum); HDB, nucleus of the horizontal limb of the diagonal band of Broca; ic, internal capsule; IPAC, interstitial nucleus of the posterior limb of the anterior commissure; LPO, lateral preoptic area; LV, lateral ventricle; MCPO, magnocellular preoptic nucleus; MPA, medial preoptic area; SI, substantia innominata; VP, ventral pallidum. Scale bar: **(A–C)** 200 μm; **(D)** 750 μm; **(E,F)** 50 μm.

Many positive cells were observed in these areas. First, a few positive cells were located near the ventral part of the lateral ventricle, in the accumbens nucleus (Figure 3A). Scattered isolated positive cells were found in the medial septal nucleus and the nucleus of the vertical limb of the diagonal band of Broca.

Different divisions of the bed nucleus of the stria terminalis presented abundant SCGN-positive cells. A group of dense-packed cells, labeled both in the somata and the neurites, was found in the medial division of this nucleus (Figures 3B–D, 5C). Some of these cells were big and with intense staining, while others were small, more rounded, and with weaker staining, but all of them were homogeneously distributed. Abundant cells with similar sizes in different parts of the lateral division of the bed nucleus of the stria terminalis were also detected (Figure 3C).

Scattered cells were found throughout the area ventral to the anterior commissure. Laterally to the third ventricle, there were many SCGN-positive cells in the ventral pallidum, the interstitial nucleus of the posterior limb of the anterior commissure, the nucleus of the horizontal limb of the diagonal band of Broca, the substantia innominata, the lateral preoptic area, and the magnocellular preoptic nucleus (Figure 3D). Throughout these nuclei, cells were dispersed and had variable morphologies, as some of them had large sizes and intense staining in the soma and the neurites, but others had a small size and weaker staining. In addition, some of them had a bipolar morphology (Figures 3D, E), while others were multipolar with irregular somas (Figures 3D, F). To conclude with the preoptic area, both in the medial preoptic area and the medial preoptic nucleus many SCGN-positive cells were present, but with faint staining (Figure 5C).

**FIGURE 4:**
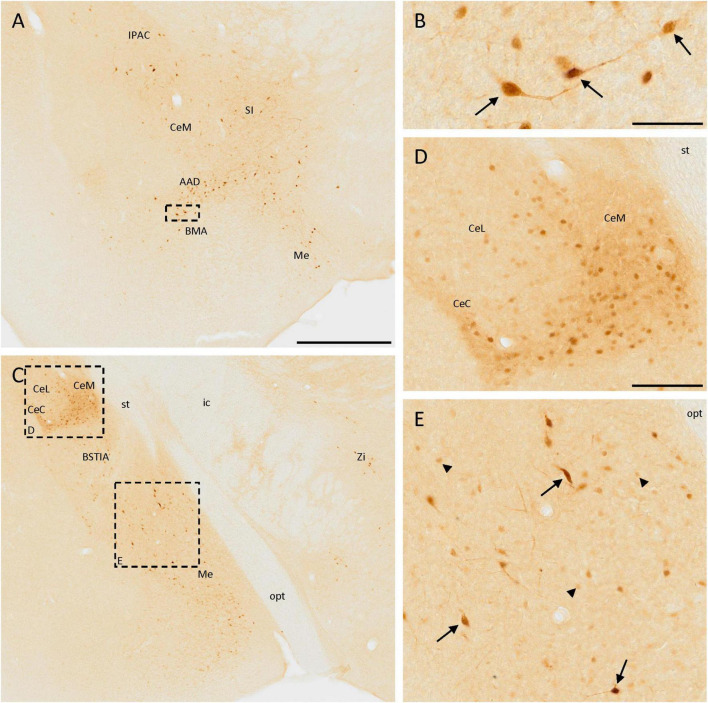
SCGN immunopositive cells in the amygdala. **(A)** SCGN-positive cells in different nuclei from the amygdala. Especially in the anterior part of the basomedial amygdaloid nucleus (BMA) or the dorsal part of the anterior amygdaloid area (AAD), the cells are big and well-stained and some of them are bipolar. **(B)** Detail of some bipolar cells found in the BMA/AAD (arrows). **(C)** Stained cells in a more caudal part of the amygdala, showing different distribution and morphology. There are also some isolated cells in the zona incerta (Zi). **(D)** Magnification of positive cells in the central amygdaloid nucleus (Ce), showing different distributions in the medial division (CeM) and capsular part (CeC) compared to the lateral division (CeL) of the nucleus. **(E)** Magnification of SCGN-positive cells with different morphologies in the medial amygdaloid nucleus (Me). Some of them were big cells strongly labeled (arrows), and others were smaller, rounded, and with faint staining (arrowheads). BSTIA, bed nucleus of the stria terminalis, intraamygdaloid division; ic, internal capsule; IPAC, interstitial nucleus of the posterior limb of the anterior commissure; opt, optic tract; SI, substantia innominata; st, stria terminalis. Scale bar: **(A,C)** 500 μm; **(B)** 50 μm; **(D,E)** 100 μm.

The distribution of SCGN immunoreactivity in the amygdala was very remarkable. In the rostralmost part of the anterior part of the basomedial amygdaloid nucleus or the dorsal part of the anterior amygdaloid area, few disperse, and strongly labeled cells were observed (Figure 4A). They had a big size and some of them had a bipolar morphology (Figure 4B). Close to these nuclei, heterogeneous immunostaining was observed in the central amygdaloid nucleus, where the expression of SCGN changed depending on the division. In the rostral part of the medial division, cells were dispersed (Figure 4A), but in more caudal parts cells were smaller, more abundant, densely packed, and with a more intense staining (Figures 4C, D). The cells found in the capsular part of the amygdaloid nucleus were like those appearing in the dorsal part of the medial division, but between these regions, in the lateral division of this nucleus, positive cell density was much lower (Figures 4C, D). The medial amygdaloid nucleus is also an area where SCGN expression was noteworthy. Here, both small cells with weak staining and bigger cells strongly labeled were observed (Figures 4A, C, E). Finally, some scattered weakly stained cells were detected in the intraamygdaloid division of the bed nucleus of the stria terminalis (Figure 4C).

### 3.4. Hypothalamus

**FIGURE 5:**
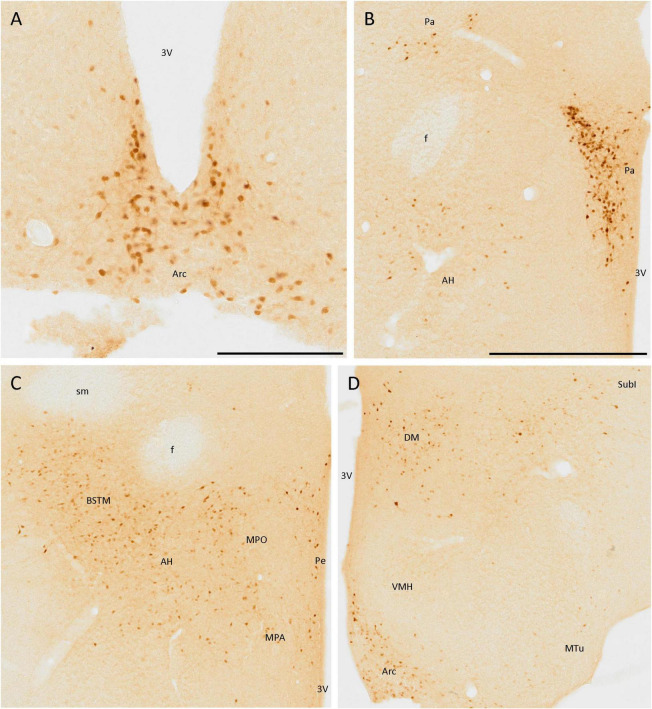
SCGN immunopositive cells in the hypothalamus **(A)** Positive cells in the arcuate nucleus (Arc), covering most of the area of the nucleus, and clearly defining its limits. **(B)** Cells in the paraventricular hypothalamic nucleus (Pa) have similar staining to that found in the Arc. Few disperse positive cells found in the anterior hypothalamic area (AH) showing faint staining. **(C)** General view of some hypothalamic nuclei and the medial division of the bed nucleus of the stria terminalis (BSTM), showing more positive cells in the AH than in B. Few dispersed cells can be observed in the periventricular hypothalamic nucleus (Pe). **(D)** Positive cells in the Arc still delineate the nucleus. In the dorsomedial hypothalamic nucleus (DM), cells with similar staining but distributed irregularly in the nucleus were observed. Few positive cells were present in the subincertal nucleus (SubI) and cells with faint staining were observed in the ventromedial hypothalamic nucleus (VMH) and the medial tuberal nucleus (MTu). 3V, third ventricle; f, fornix; MPA, medial preoptic area; MPO, medial preoptic nucleus; sm, stria medullaris of the thalamus. Scale bar: **(A)** 200 μm; **(B–D)**, 500 μm.

Secretagogin was present in many hypothalamic nuclei, with different cell populations differing in size, staining, and area of the nuclei. The staining was very remarkable in the arcuate nucleus, as positive cells were present in all the nuclei and clearly defined their limits (Figures 5A, D). These cells were mainly rounded, and the staining was stronger in the somata than in the neurites. Something similar happened in the paraventricular hypothalamic nucleus, where positive cells were very abundant but, in this case, neurites were also strongly labeled (Figure 5B).

Apart from these nuclei, SCGN expression was observed in other areas, but it did not define the limits of nuclei as well as in the above-mentioned cases. Some positive cells with low-intensity labeling were detected in the anterior hypothalamic area (Figures 5B, C), the ventromedial hypothalamic nucleus, and the medial tuberal nucleus (Figure 5D). Finally, many SCGN-positive cells were found in the dorsomedial hypothalamic nucleus, but their distribution within it was quite heterogeneous, as they were very abundant in some areas and almost absent in others (Figure 5D).

The periventricular hypothalamic nucleus also had some isolated SCGN-positive cells (Figure 5C), but their number was small. Few, weakly labeled cells were observed in the subincertal nucleus (Figure 5D).

### 3.5. Diencephalon

**FIGURE 6:**
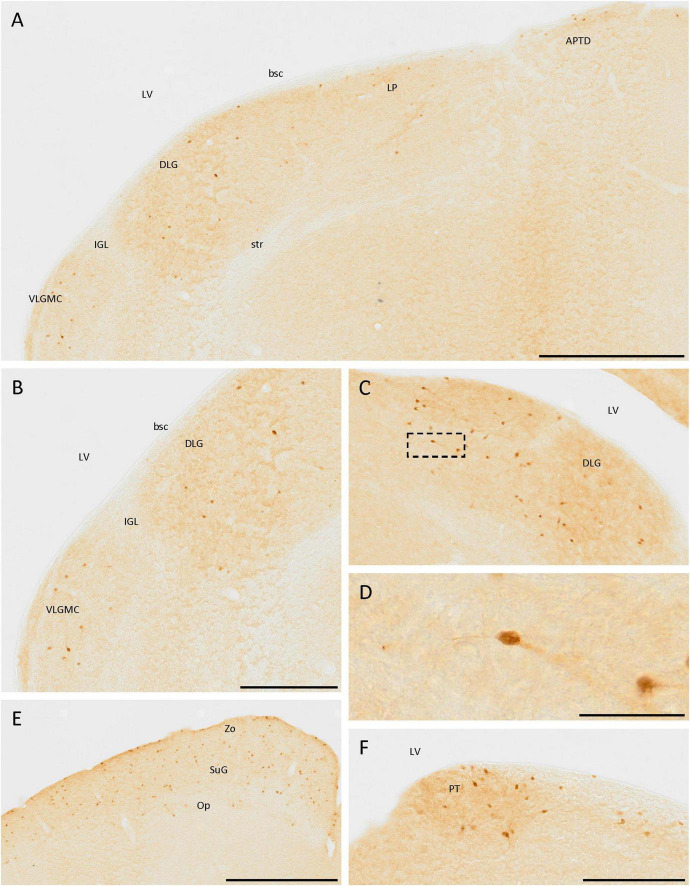
SCGN-positive cells in the diencephalon and mesencephalon. **(A)** Cells expressing SCGN from the magnocellular part of the ventral lateral geniculate nucleus (VLGMC) to the anterior pretectal nucleus, dorsal part (APTD), mainly in the most dorsal part of the nuclei. **(B)** Higher magnification of cells in the VLGMC and the dorsal lateral geniculate nucleus (DLG). **(C)** Detail of the DLG nuclei, where most of the positive thalamic neurons were detected, some of them showing a bipolar morphology. **(D)** Detail of a bipolar cell in the DLG [boxed in panel **(C)**]. **(E)** SCGN-positive cells in the different layers of the superior colliculus. **(F)** Group of positive cells in the pretectal zone (PT). bsc, brachium of the superior colliculus; IGL, intergeniculate leaf; LP, lateral posterior thalamic nucleus; LV, lateral ventricle; Op, optic nerve layer of the superior colliculus; str, superior thalamic radiation; SuG, superficial gray layer of the superior colliculus; Zo, zonal layer of the superior colliculus. Scale bar: **(A)** 500 μm; **(B,C)**, 250 μm; **(D)** 50 μm; **(E)** 500 μm; **(F)** 200 μm.

In the thalamus, cells expressing SCGN were observed in the dorsal lateral geniculate nucleus and the magnocellular part of the ventral lateral geniculate nucleus (Figures 6A–C). The cells were more abundant in the first than in the second one, where they were more frequent at the border with the ventricle. Most cells had less intense staining in the neurites than in the soma, but in both cases, bipolar cells were observed (Figures 6C, D). Apart from the lateral geniculate nucleus, some immunopositive cells were detected in the dorsal part of the lateral posterior thalamic nucleus (Figure 6A). These cells were close to the ventricle, along the brachium of the superior colliculus from the dorsal lateral geniculate nucleus to the dorsal part of the anterior pretectal nucleus (Figure 6A). In the pretectal zone, close to the ventricle, many positive cells were found in a well-defined group (Figure 6F).

In the prethalamus, some weakly labeled cells were observed in the zona incerta (Figure 4C).

### 3.6. Mesencephalon

There were different parts in the mesencephalon containing SCGN-positive cells. Of these, the nucleus that showed the highest expression was the superior colliculus, specifically the superficial gray and the zonal layer, with less cells in the optical nerve layer (Figure 6E). In the substantia nigra, labeled cells were also observed, but the number was small (data not shown).

### 3.7. Brainstem and cerebellum

**FIGURE 7:**
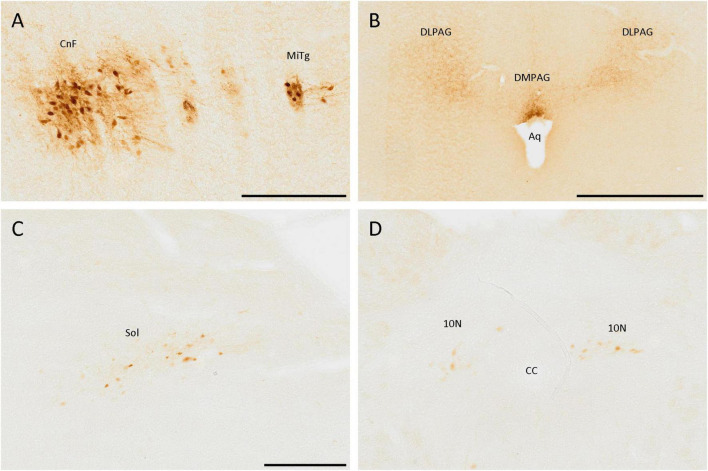
SCGN-positive cells in the brainstem. **(A)** SCGN-positive cells and neurites strongly labeled in the cuneiform nucleus (CnF) and microcellular tegmental nucleus (MiTg). **(B)** Fibers in the dorsolateral and dorsomedial periaqueductal gray (DLPAG and DMPAG, respectively), with more intense staining closer to the aqueduct (Aq). **(C)** Disperse positive cells in the nucleus of the solitary tract (Sol). **(D)** Positive cells in the dorsal motor nucleus of the vagus (10N). CC, central canal. Scale bar: **(A)** 200 μm; **(B)** 500 μm; **(C,D)** 250 μm.

The brainstem also had cell populations positive for SCGN. The most remarkable was the one in the most ventral part of the cuneiform nucleus, formed by a dense group of adjacent cells with many strongly labeled neurites (Figure 7A). Close to the cuneiform nucleus, in a lateral direction, a small population, constituted mainly by fibers and a few cells, was also detected, which belong to the microcellular tegmental nucleus (Figure 7A).

Regarding neurites, there were many of them around the aqueduct, mainly in the dorsolateral and dorsomedial periaqueductal gray (Figure 7B).

In more caudal zones, a SCGN-immunoreactive cell population was detected in the nucleus of the solitary tract, formed by big, well-localized, and faintly labeled cells (Figure 7C). Nevertheless, this population was only present in the anterior part of the nucleus. In more posterior zones, similar cells were observed in the dorsal motor nucleus of the vagus (Figure 7D). The cerebellum was devoid of SCGN-positive cells.

**FIGURE 8:**
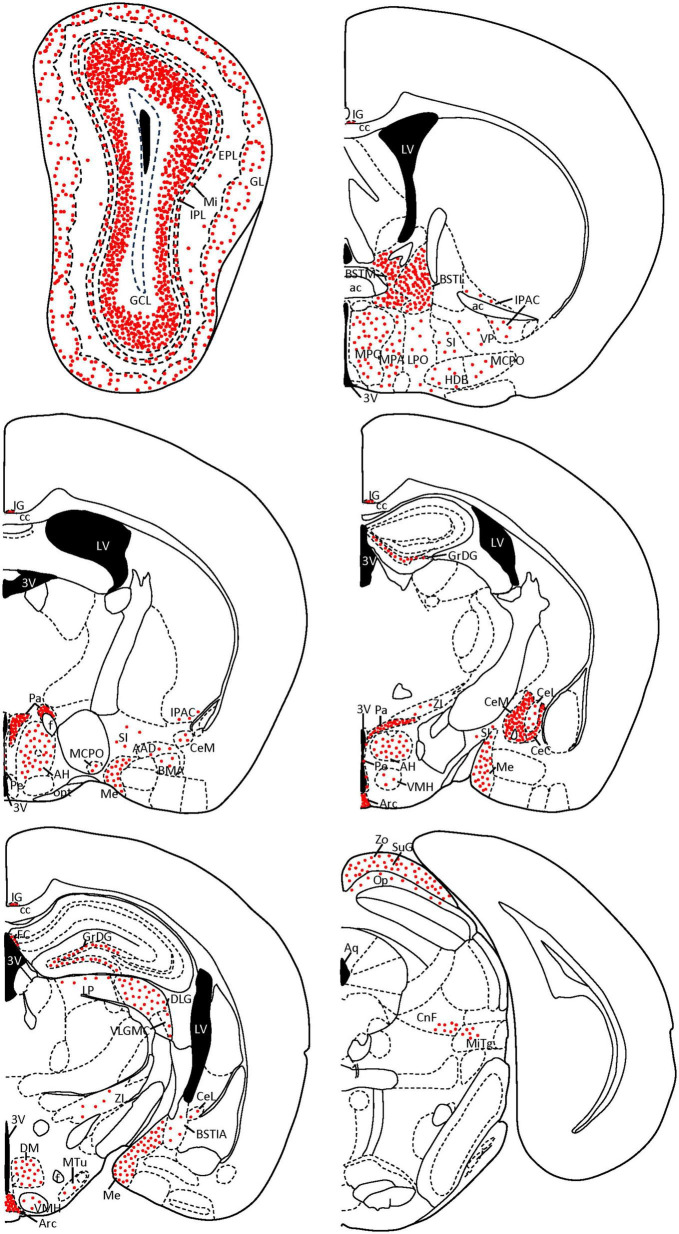
Coronal diagrams of SCGN expression in the mouse brain. The number of red dots is related to the quantity of SCGN-positive somata found on each nucleus. Dots follow the distribution patterns found in each nucleus (e.g., cell somata are arranged in circles in the GL of the olfactory bulb, while in the GCL they cover the external part of the layer, top left scheme). Based on Paxinos and Franklin (2001). 3V, third ventricle; AAD, anterior amygdaloid area, dorsal part; ac, anterior commissure; AH, anterior hypothalamic area; Aq, aqueduct; Arc, arcuate nucleus; BMA, basomedial amygdaloid nucleus, anterior part; BSTIA, bed nucleus of the stria terminalis, intraamygdaloid division; BSTL, bed nucleus of the stria terminalis, medial division; BSTM, bed nucleus of the stria terminalis, medial division; cc, corpus callosum; CeC, central amygdaloid nucleus, capsular part; CeL, central amygdaloid nucleus, lateral division; CeM, central amygdaloid nucleus, medial division; CnF, cuneiform nucleus; DLG, dorsal lateral geniculate nucleus; DM, dorsomedial hypothalamic nucleus; EPL, external plexiform layer; f, fornix; FC, fasciola cinereum; GCL, granule cell layer; GL, glomerular layer; GrDG, granule cell layer of the dentate gyrus; HDB, nucleus of the horizontal limb of the diagonal band of Broca; IG, indusium griseum; IPAC, interstitial nucleus of the posterior limb of the anterior commissure; IPL, internal plexiform layer; LP, lateral posterior thalamic nucleus; LPO, lateral preoptic area; LV, lateral ventricle; MCPO, magnocellular preoptic nucleus; Me, medial amygdaloid nucleus; Mi, mitral cell layer; MiTg, microcellular tegmental nucleus; MPA, medial preoptic area; MPO, medial preoptic nucleus; MTu, medial tuberal nucleus; Op, optic nerve layer of the superior colliculus; opt, optic tract; Pa, paraventricular hypothalamic nucleus; Pe, periventricular hypothalamic nucleus; SI, substantia innominata; SuG, superficial gray layer of the superior colliculus; VLGMC, ventral lateral geniculate nucleus, magnocellular part; VMH, ventromedial hypothalamic nucleus; VP, ventral pallidum; ZI, zona incerta; Zo, zonal layer of the superior colliculus.

A summary of the results is shown in Table 1 and Figure 8.

## 4. Discussion

Several studies have analyzed the expression of SCGN in different brain areas of mice (Mulder et al., 2010; Gyengesi et al., 2013; Kosaka and Kosaka, 2013, 2018), but none of them has studied the whole brain, leaving many regions undescribed until the present study. In addition, many of these studies were carried out in developing or young animals, while we have used adult mice. Multiple studies have shown that the expression of different calcium-binding proteins changes during development (Hendrickson et al., 1991; Alfahel-Kakunda and Silverman, 1997; Fujiwara et al., 1997), so the use of adult mice can supplement those results from developing animals and provide a broader view of SCGN expression. The Allen Brain Atlas for mouse provides *Scgn* gene expression by *in situ* hybridization^1^ (Lein et al., 2007) and the areas where the expression levels are higher (such as the different layers of the olfactory bulb) perfectly agree with those where we found more positive cells, which makes our results more robust.

Secretagogin is widely distributed in the mouse brain, similarly like other sensor calcium-binding proteins. This is not surprising, as Ca^2+^ homeostasis has to be finely regulated (Braun, 1990). However, SCGN distribution in mice is different from other calcium-binding proteins, and its total absence in the cerebellum and many cortical areas is really striking. Comparing our results in mice with those previously obtained in the rat brain, in both animal models SCGN-positive cells are missing in the cerebellum (Maj et al., 2012). In addition to this, similarities in SCGN expression can be found in the olfactory bulb, amygdala, hippocampus, basal ganglia, paraventricular nucleus or the superior colliculus of both species (Maj et al., 2012; Tapia-González et al., 2020; Hevesi et al., 2021). On the other hand, we did not find SCGN-positive cells in the supraoptic or suprachiasmatic nuclei, nor in the caudate putamen or the habenular region in mice, although its presence has been described in these nuclei in the rat brain (Maj et al., 2012).

Secretagogin expression is abundant in areas like the olfactory bulb and the hippocampus not only in rodents, but also in other mammals, such as humans (Attems et al., 2012; Tapia-González et al., 2020), red foxes (Ortiz-Leal et al., 2023), and even in chickens (Gáti et al., 2014) and catfishes (Basu et al., 2022). Knowing that the olfactory bulbs are part of the archicortex (the most primitive domain of the cerebral cortex), and that the hippocampus is included in the paleocortex, the next oldest cortical region, SCGN could have appeared in the brain early in evolution and could remain present in these cortical areas but not in more recent ones, where other calcium-binding proteins are abundant. A preserved role of SCGN in evolution has been also suggested by other authors (Gáti et al., 2014). On the other hand, some studies have compared the expression of SCGN in different mammals, mainly in mice, rats and humans, showing differences in the pattern of expression in areas like the developing neocortex (Raju et al., 2018), the hippocampus (Tapia-González et al., 2020) or the cerebellum (Maj et al., 2012), which shows phylogenetic differences across mammalian species, as has been previously reported (Mulder et al., 2010). Even though it is difficult to identify the cause for these differences, we could think that SCGN is not playing an essential role if it is present in some animals but not in others. Further research is necessary to know the specific function of SCGN in different animal species.

The expression of SCGN is also very heterogeneous when comparing different cerebral nuclei. Some nuclei had many SCGN-positive cells, which makes SCGN an ideal marker to identify them. This particular distribution could be related to the fact that having the same protein in most of the cells allows the modulation of the firing rate, as it happens with PV in the reticular thalamic nucleus (Albéri et al., 2013). By contrast, other nuclei had few SCGN-positive cells, which may mean that they express other calcium-binding proteins and that these cells have different functions. It has been observed that, in rat and primate striatum, SCGN separates two PV-positive cell populations with different structures, physiology, and topography (Garas et al., 2016). Future research combining SCGN with different calcium-binding proteins in mice is needed to confirm if these different distributions can be related to distinct functions. However, the colocalization analysis is beyond the scope of this work on the SCGN distribution.

Secretagogin might be a key element for the correct functioning of the senses, as it has been suggested by other authors due to its distribution in different sensory brain areas (Maj et al., 2019). Indeed, in the olfactory system, SCGN is present in a great number of cells of the olfactory bulb, but it also appears in the amygdala and other nuclei related to olfaction, such as the magnocellular preoptic nucleus and the horizontal limb of the diagonal band (Záborszky et al., 1986). This reinforces the idea that SCGN plays an essential role in olfaction, as previously described (Kosaka and Kosaka, 2013; Gáti et al., 2014; Pérez-Revuelta et al., 2020). SCGN expression is also remarkable in the visual system as immunoreactive cells are present in the superior colliculus and lateral geniculate nucleus, as well as in different cells of the retina (Puthussery et al., 2010; Weltzien et al., 2014). Finally, SCGN might be related to the sense of touch, as it appears in the cuneiform nucleus, the area where the secondary neurons of the dorsal column-medial lemniscus pathway (which carries the information from the posture, vibratory sensitivity, and discrimination between two points) are located. In addition, some authors have related SCGN expression to neurons that modulate nociception in the spinal cord of mice, rats, and humans (Shi et al., 2012).

Finally, SCGN may play an important role as a regulatory and neuroprotective protein at the cellular level. It acts as a regulatory protein in the amygdala (Hevesi et al., 2021) and controls the release of the stress hormone in the paraventricular hypothalamic nucleus (Romanov et al., 2015). Given the high density of SCGN-positive cells in the hypothalamus, it may also control the release of other hormones, such as the growth hormone, since it is released from the arcuate nucleus, where the SCGN expression was very abundant and similar to that found in the paraventricular hypothalamic nucleus. Finally, it is known that different calcium-binding proteins can influence the susceptibility of neurons to neurodegeneration (Fairless et al., 2019), and considering the expression of SCGN in the hippocampus, it is not surprising that it plays a role in neuronal resistance in Alzheimer’s disease (Attems et al., 2008). The neuronal resistance may be related to the type of damage, as SCGN expression increases in olfactory-deprived mice, but it does not change in the PCD (Purkinje Cell Degeneration) mice, which show neurodegeneration of the olfactory bulb (Pérez-Revuelta et al., 2020).

From these results, we can conclude that SCGN is widely distributed in the adult mouse brain and could be used as a specific marker for certain nuclei. SCGN is expressed by different neuronal populations and, based on their distribution and morphology, they might be involved in several functions and with different specific roles. Therefore, we consider the study of SCGN to be a very promising field that can help us to properly understand the regulatory systems of Ca^2+^ levels in the CNS, as well as other interesting neuronal properties, such as neuroresistance or vulnerability.

## Data availability statement

The original contributions presented in this study are included in the article/supplementary material, further inquiries can be directed to the corresponding author.

## Ethics statement

The animal study was approved by the Bioethics Committee of the University of Salamanca (reference number: #00613). The study was conducted in accordance with the local legislation and institutional requirements.

## Author contributions

PT, DD, and JA conceived the study, designed the experiments, wrote the manuscript, and prepared the figures. PT, LP-R, ÁC-A, and CH-P performed the histological analysis. PT, JV, TC, and EW carried out the image analyses. PT was a major contributor to the writing of the manuscript and the organization and design of all the figures. All authors critically revised and approved the final manuscript.

## Footnotes

1
http://mouse.brain-map.org/gene/show/84639
